# Alterations in Expression and Function of ABC Family Transporters at Blood-Brain Barrier under Liver Failure and Their Clinical Significances

**DOI:** 10.3390/pharmaceutics10030102

**Published:** 2018-07-23

**Authors:** Yilin Fan, Xiaodong Liu

**Affiliations:** Center of Drug Metabolism and Pharmacokinetics, China Pharmaceutical University, Nanjing 210009, China; fanyilin0109@stu.cpu.edu.cn

**Keywords:** blood-brain barrier, liver failure, P-glycoprotein, breast cancer resistance protein, multidrug resistance-associated proteins, hepatic encephalopathy

## Abstract

Liver failure is often associated with hepatic encephalopathy, due to dyshomeostasis of the central nervous system (CNS). Under physiological conditions, the CNS homeostasis is precisely regulated by the blood-brain barrier (BBB). The BBB consists of brain microvessel endothelial cells connected with a junctional complex by the adherens junctions and tight junctions. Its main function is to maintain brain homoeostasis via limiting the entry of drugs/toxins to brain. The brain microvessel endothelial cells are characterized by minimal pinocytotic activity, absent fenestrations, and highly expressions of ATP-binding cassette (ABC) family transporters (such as P-glycoprotein, breast cancer resistance protein and multidrug resistance-associated proteins). These ABC transporters prevent brain from toxin accumulation by pumping toxins out of brain. Accumulating evidences demonstrates that liver failure diseases altered the expression and function of ABC transporters at The BBB, indicating that the alterations subsequently affect drugs’ brain distribution and CNS activity/neurotoxicity. ABC transporters also mediate the transport of endogenous substrates across the BBB, inferring that ABC transporters are also implicated in some physiological processes and the development of hepatic encephalopathy. This paper focuses on the alteration in the BBB permeability, the expression and function of ABC transporters at the BBB under liver failure status and their clinical significances.

## 1. Introduction

**Figure 1:**
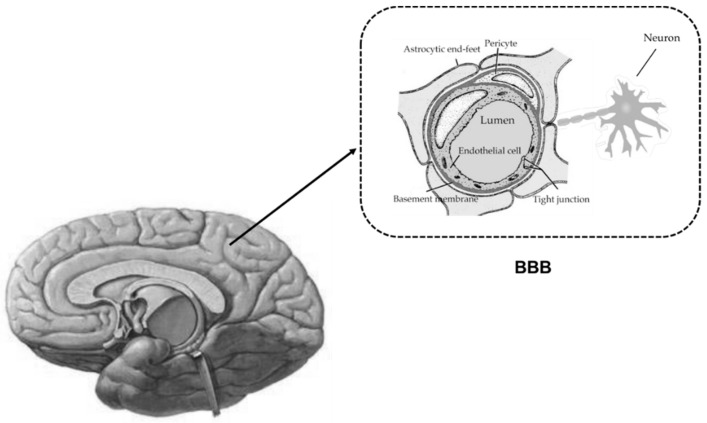
Cellular interfaces between blood and brain parenchyma (blood-brain barrier, BBB). The endothelial cells and the closely apposed pericytes are completely unsheathed by overlapping astrocytic end-feet and neuronal terminals, forming “neurovascular unit” (NVU).

Liver failure is often associated with hepatic encephalopathy, due to accumulation of neurotoxins in brain. Under physiological conditions, the homeostasis of central nervous system (CNS) is precisely regulated by the blood–brain barrier (BBB). Serving as an interface for communication between CNS and blood circulation, the BBB is crucial in limiting free diffusion between brain fluids and blood. This provides transport processes for essential nutrients, ions, signaling molecules and metabolic waste products. The BBB consists of brain microvessel endothelial cells connected with a junctional complex by the adherens junctions and tight junctions. In addition, multiple transporters, receptors and enzymes are highly expressed at the BBB. Thus, the BBB is not only an anatomical barrier, but also a dynamic tissue. The primary anatomic units of the BBB are brain capillaries, where the endothelial cells of brain capillaries and the closely apposed pericytes are completely unsheathed by overlapping astrocytic end-feet, microglia and neuronal terminals, termed as a “neurovascular unit” (Figure 1) [1].

Compared to peripheral vasculature, endothelial cells of brain capillaries are characterized by minimal pinocytotic activity, absent fenestrations and high expression of tight junction proteins at cell-cell contacts [1,2]. Tight junctions consist of multiple proteins, and include junctional adhesion molecules(JAMs), occludin, claudins (such as claudin-1, claudin-3, and claudin-5), and zonulla occluden proteins (such as ZO-1, ZO-2 and ZO-3) [2]. They form the primary physical barrier component of the BBB, and their function is to restrict paracellular entry of various endogenous and exogenous substances—leading to higher transendothelial electrical resistance (1500~2000 Ωcm^2^) across the BBB compared with that (i.e., 3–33 Ωcm^2^) in other vascular tissues [1,2].

About 99% of cerebral capillaries are covered by astrocyte end-feets. The critical cell-cell interactions directly modulate the BBB’s characteristics by secreting some factors, such as transforming growth factor-β (TGF-β), glial cell line-derived neurotrophic factor (GDNF), and basic fibroblast growth factor (BFGF). Pericytes are attached to the abluminal side of brain capillary endothelial cells and to the luminal side of the astrocyte end feet, which contribute not only to vascular contractility and immune responses but also to BBB functional integrity. It was reported that the percentage of cerebral capillaries covered by pericytes positively correlated with “tightness” of the junctions between endothelial cells and inversely correlated with BBB permeability [2,3]. Pericytes are considered to be essential for proper localization of endogenous BBB proteins and induction of BBB properties [4,5]. In addition, the extracellular matrix regulates the expression of tight junction proteins and BBB integrity. Disruption of the extracellular matrix was reported to be associated with the loss of the BBB function and the increases in BBB permeability [2].

Both brain microvessel endothelial cells and associated astrocyte processes are distinctly connected with noradrenergic, serotonergic, cholinergic and GABAergic neurons, demonstrating that BBB function is probably regulated by these neurons. Loss of direct noradrenergic input from the locus coeruleus was reported to increase BBB susceptibility to acute hypertension, with a significant increase in BBB permeability to [^125^I]-albumin [6]. Furthermore, stimulation of the postganglionic parasympathetic fibers of the sphenopalatine ganglion also increased vascular leakage as well as the levels of anti-HER2 monoclonal antibody and etoposide in brain [7].

In general, hepatic encephalopathy is considered to be the main cause of accumulation of brain neurotoxins due to BBB dysfunctions (i.e., liver-brain axis) [8,9,10]. A report showed that efflux of phenylalanine from brain to blood significantly decreased in patients with hepatic encephalopathy, leading to a significant increase in brain interstitial fluid phenylalanine concentration [9]. The extent of alterations was positively correlated to coma grade [9]. Quinolinic acid is also an endogenous neuroexcitant derived from tryptophan. It was found that quinolinic acid levels in brain of patients who died of acute liver failure were significantly elevated [10]. Rat experiment showed that elevated levels of brain quinolinic acid were strongly correlated with fulminant hepatic failure [11]. These results indicate that quinolinic acid is involved in pathogenesis of hepatic encephalopathy.

## 2. ABC Drug Transporters Expressed at BBB and Their Functions

**Figure 2:**
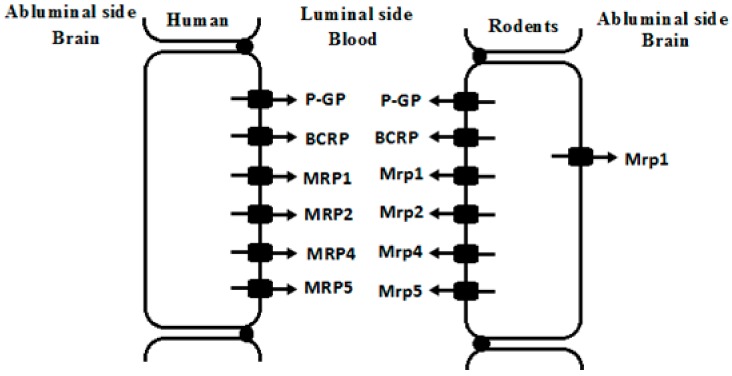
Possible location of some ATP-binding cassette (ABC) drug transporters in brain microvessel endothelial cells of human and rodents. P-GP, glycoprotein; BCRP, breast cancer resistance protein; MRPs/Mrps, multidrug associated resistance proteins.

Endothelial cells of brain capillaries highly express ATP-binding cassette (ABC) transporters and solute carriers (SLC), constructing BBB components. The SLC transporters expressed at the BBB include organic anion transporting polypeptides (OATPs), organic anion transporters (OATs) and organic cation transporters (OCTs). These SLC transporters are usually located in the luminal or abluminal membranes of the endothelial cells and facilitate drug transport across BBB. Here, we focus on the alterations in the expression and function of brain ABC transporters under liver failure conditions. ABC transporters expressed at brain include P-glycoprotein (P-GP/ABCB1), breast cancer resistance protein (BCRP/ABCG2), and multidrug associated resistance proteins (MRPs/ABCCs). These ABC transporters are mainly located in the luminal membranes of the endothelial cells (Figure 2) and efflux drugs out of CNS [2,3].

P-GP, coded by ABCB1 gene, is mainly located in the luminal membranes of brain microvessel endothelial cells, playing the function of protecting CNS from exposure to potential neurotoxins and maintaining the homeostasis [12,13]. The importance of P-GP in CNS protection has been highlighted in Abcb1-/- mice [14,15,16,17]. It was found that Abcb1b-/- mice showed a 100-fold increase in brain uptake of ivermectin, a neurotoxic pesticide, leading to a significant increase in ivermectin toxicity compared with wild-type mice [17]. Abcb1-/- mice showed a nine-fold higher brain concentration of asimadoline, and an eight-fold more sensitivity to the sedative effect of asimadoline compared with wild-type mice [15]. The relationship between increases in brain penetration and Abcb1 deficiency was also demonstrated in other drugs such as amiodarone, loperamide, quinidine, verapamil and digoxin [14,16].

P-GP possesses a broad spectrum of substrates, such as: Antibiotics, calcium channel blockers, cardiac glycosides, chemotherapeutics, immunosuppressant, anti-epileptics, HIV-1 protease inhibitors, and some toxins. In addition, P-GP is also involved in the transport of some drug-delivery vectors (i.e., carbon allotropes) [18]. Thus P-GP expressed at the BBB becomes a formidable obstacle to CNS drug delivery?limiting the ability of effective treat for CNS disorders. P-GP also transports some endogenous substrates, including cytokines, lipids, steroid hormones, and peptides [12,13], which shows its physiological roles. Some diseases alter the expression of P-GP at BBB. Clinical trials and animal experiments have demonstrated that Alzheimer’s disease (AD) significantly downregulated the expression and function of P-GP at brain [19,20,21,22]. Some peripheral diseases, such as diabetes [23,24,25] and liver failure [26,27,28], also downregulated brain P-GP expression and function—enhancing pharmacological effect on CNS. Conversely, overexpression of P-GP also occurred in capillary endothelial cells of epilepsia patients [29] or epilepsia animals [30]. Some antiepileptic drugs are P-GP substrates, indicating that P-GP overexpression at the BBB contributes to the pharmacoresistance observed in epilepsia patients [30,31].

BCRP, another important efflux transporter coded by *ABCG2* gene, is predominantly expressed at the luminal membrane of microvessel endothelial cells, and protects brain from the toxicity of xenobiotics. Tyrosine kinase inhibitors (such as gefitinib, imatinib, nilotinib, erlotinib, lapatinib, and sunitinib), methotrexate, other drugs (such as prazosin, elacridar, and mitoxantrone), or some endogenous compounds (such as dehydroepiandrosterone sulfate and urate) are BCRP substrates. The BCRP expression at the BBB also impedes these drugs to reach their intracerebral targets for treating CNS disorder, such as brain tumors.

It is noteworthy that in vitro BCRP functional analysis using cell lines overexpressing BCRP may not often accurately reflect in vivo BCRP function. For example, in vitro data demonstrated that some compounds (cimetidine, alfuzosin, dipyridamole, LY2228820, dehydroepiandrosterone sulfate, and mitoxantrone) were substrates of BCRP, but Abcg2 deficiency slightly affected brain uptakes of these compounds in mice [32,33]. At the BBB, BCRP is co-located with P-GP, and also share substrates with P-GP: Indicating that the two transporters work together to limit xenobiotics entering into the brain. In vitro study demonstrated that BCRP contributed more efficient transport of vemurafenib than P-GP, but Abcg2 deficiency did not increase brain distribution of vemurafenib. However, Abcb1 deficiency still significantly increased brain distribution of vemurafenib (2.9-fold). Importantly, both Abcb1 and Abcg2 knockout resulted in remarkable 80-fold increase in brain distribution of vemurafenib [34], demonstrating the “synergistic” role of the two transporters at the BBB. The “synergistic” effect was also found in other drugs such as dasatinib [35], elacridar [36],erlotinib [37], gefitinib [38], rucaparib [39], sorafenib [40], and topotecan [41]. Cooperation of P-GP with BCRP implies that the absence of either P-GP or BCRP alone does not appreciably increase brain penetration of these dual substrates, and the greatest enhancement in brain penetration of dual substrates is always seen when both P-GP and BCRP are absent. Accordantly, dual P-GP/BCRP inhibitor elacridar showed the strongest enhancement of brain distribution of gefitinib although P-GP inhibitor (LY335979) or BCRP inhibitor (Ko143) alone mildly altered brain distribution of gefitinib [38].

MRPs, coded by ABCC gene, also extrude xenobiotics from brain. Substrates of MRPs are generally organic anions and their glucuronidated, sulfated, and glutathione-conjugated metabolites. However, the expression and distribution of MRPs at the BBB often show controversial reports. For example, MRP1, MRP2, MRP3, MRP4 and MRP5 mRNA were detected in brain samples of human, among which expression of MRP5 mRNA showed the highest expression. However, MRP1, MRP2, MRP3, and MRP4 mRNA showed considerable variation. Immunofluorescence analysis showed that MRP1, MRP4 and MRP5 were primarily localized in the luminal membrane of capillary endothelial cells, but no reactivity for the MRP2 or MRP3 proteins [42]. Human brain microvascular endothelial cells exhibited the strongest expression of MRP1 mRNA, followed by MRP4, MRP5 and MRP6, but MRP2 and MRP3 showed very weak expression [43].

Expression of MRPs also shows large species variations. A report on five species (human, rat, mouse, pig and cow) showed that mRNA levels of MRP1/Mrp1 were detected to be high in brain microvessel endothelial cells of human, rat and cow, low in mouse, and negligible in pig. MRP3/Mrp3 expressions in mouse and pig were higher than other species. The highest expression of MRP4/Mrp4 was found in rodents, followed by pig and human. MRP5/Mrp5 was highly expressed in brain endothelial cells of human, rat, pig, and cow, with lower expression in mice. No significant expression of MRP2/Mrp2 was detected in the brain of indicated species (Table 1) [44]. Mrp2 expression in brain of mice is dependent on strain. For example, FVB mice lacked Mrp2 in the brain vascular and choroid plexus endothelium although Mrp2 was still present in liver and kidney of the strain, indicating that FVB mice represented a spontaneous, brain-specific “knockout” of Mrp2 [45].

Cystic fibrosis transmembrane conductance regulator (CFTR), belonging to ABCC family (ABCC7), is also exclusively expressed in neurons of adult and neonatal human brains [46,47] as well as rat brains [48]. It was found that CFTR expression in developing cerebral cortex was increased progressively during development although faint expression was observed at early stage. Moreover, cystic fibrosis patient (p.F508del mutation) showed also very weak expression of brain CFTR, which was consistent with a slight delay in maturation, especially at the level of the neuroepithelium [46]. These results indicate that CFTR is involved in neuronal maturation and differentiation.

## 3. Alterations in BBB Permeability under Liver Failure

Liver failure, especially acute liver failure (ALF), is often accompanied by hepatic encephalopathy, brain edema and hepatic coma. Brain edema and hepatic coma are lethal. The incidence of brain edema in patients with hepatic failure was reported to reach 51.4% [49]. The mechanisms of brain edema are generally categorized as cytotoxic and vasogenic. Accumulation of water and plasma constituents in the extracellular region was attributed to alteration of structural BBB [49], although ultrastructural alterations in the BBB were slightly changed. It was found that in cerebral cortex obtained immediately after death from nine patients with acute liver failure, the intercellular tight junctions were intact except for being slightly widened in two patients. However, the endothelial cells and perivascular astroglial foot were swollen, accompanied by increased vesicles and vacuoles [50]. ALF rabbits also demonstrated the swelling of perivascular astroglial foot and the intact tight junctions [51]. In ALF mice induced by d-galactosamine and liposaccharide, it was found that increased BBB permeability by ALF was partly attributed to disruption of tight junctions and loss of the tight junction-associated protein occluding, which might be involved in both vasogenic and cytotoxic mechanisms [52]. Our previous study also demonstrated intercellular tight junctions were intact in thioacetamide-induced ALF rats, without leakage of Evans blue into brain and alteration of the expression of tight junction proteins (claudin-5 and occluding) [26,53]. These results indicate that a vasogenic brain edema results from subtle modifications of tight junctional proteins without an obvious disruption of the BBB. However, chronic liver failure obviously disrupted BBB, leading to a significant increase of Evans blue extravasation into brain [27].

Liver failure is often associated with hyperammonemia, which is attributed to hepatic encephalopathy and brain edema. Several reports showed that arterial ammonia level directly correlated to the risk of impending brain herniation [54,55]. Ammmonia was reported to increase paracellular permeability of BBB partly via encompassing oxidative/nitrosative stress and activating matrix metalloproteinases [56]. Clinical trials [57] and animal experiments [53,58] have showed that liver failure also significantly increased some inflammatory molecules including cytokines interleukin-1 (IL-1), interleukin-6 (IL-6), and tumor necrosis factor-alpha (TNF-α) in plasma. Levels of TNF-α in plasma were reported to positively correlate with the severity of hepatic encephalopathy [59] or with the intracranial pressure [60]. Selective deletion of cellular receptors of IL-1 and TNF-α attenuated the development of encephalopathy and brain edema in experimental ALF [61]. Contrastingly, an injection of TNF-α into CNS increased BBB permeability in a dose-dependent manner [62]. Furthermore, the anti-TNF-α antibody could inhibit the increase of BBB permeability and block the entering of bacterium to CNS [62,63]. Similarly, TNF-α antibody or TNF-α-R1 antibody increased the expression of tight junction protein (occludin and ZO-1), and decreased the concentration of Evans Blue in brains of ALF mice induced by D-galactosamine and lipopolysaccharide [62,63]. In acetaminophen-induced ALF mice, it was found that increases in BBB permeability positively correlated with elevated serum TNF-α levels, which could be prevented by administering anti-TNFα-IgG [63].

Matrix Metalloproteinases (MMPs), especially, MMP-9, play an important role in numerous physiologic and pathologic processes, via digesting capillary endothelial constituents and tight junction proteins of the BBB. Brain edema and tight junction protein degradation in brain of azoxymethane-induced ALF mice were associated with increase in circulating MMP-9 levels. Blocking MMP-9 with either GM6001 or MMP-9 monoclonal antibody significantly attenuated brain extravasation, astrocytic end foot swelling, and brain edema [64]. Moreover, liver failure also increased circulating transforming growth factor β1 (TGFβ1), further promoting endothelial cell permeability. In azoxymethane-treated mice, it was reported that an administration of neutralizing antibodies against TGFβ significantly reduced BBB permeability. In vitro data also demonstrated that treatment of bEnd.3 cells with recombinant transforming growth factor β1(rTGFβ1) dose-dependently increased MMP9 expression and suppressed claudin-5 expression [65]. Furthermore, MMP-9 downregulated occludin expression via inducing transactivation of epidermal growth factor receptor (EGFR) and p38 mitogen-activated protein kinase/nuclear factor-kappa B (MAPK/NFκB) signals [66].

The increase in BBB permeability was also associated with vascular remodeling due to up-regulation of vascular endothelial growth factor (VEGF) and its receptors [67]. VEGF is known to play a main role in vascular permeability by activating MMPs. The activations of MMPs, especially MMP-9, increased BBB permeability via breaking vessel walls and degrading tight junctions [68,69,70]. Systemic and cerebral VEGF levels were significantly elevated under experimental ALF, commonly contributing to the chronic impairment of the BBB [71].

## 4. Alterations in Expression and Function of ABC Transporters at BBB by Liver Failure

Accumulating evidence [26,27,28,53,72,73,74,75] has demonstrated that liver failure altered the expression and function of ABC transporters at the BBB, and that the alterations were dependent on types of the developed liver failure and the species of ABC transporters. ALF rats were developed using intraperitoneal injection of thioacetamide (TAA) (300 mg/kg) for two days with a 24-h interval [26,53]. The functions of P-GP, BCRP and Mrp2 at rat brain were represented as brain-to-plasma ratios of their substrate (rhodamine 123 and vincristine for P-GP, prazosin and methotrexate for BCRP, dinitrophenyl-S-glutathione and sulfobromophthalein for MRP2) levels, respectively. Expressions of these transporter proteins were measured using Western blotting. The results showed that ALF significantly increased brain-to-plasma ratios of both P-GP and BCRP substrates. Concordantly, expressions of P-GP and BCRP protein in brain of ALF rats were significantly suppressed. Data from the leakage of Evans blue and expression of tight junctional protein (occludin and claudin-5) demonstrated that BBB integrity of ALF rats was intact. These results indicated that the increased distributions of the P-GP and BCRP substrates in brain of ALF rats were mainly attributed to the reduction of P-GP and BCRP function and expression [26,53]. Being different from P-GP and BCRP, the expression and function of Mrp2 at brain of ALF rats were significantly upregulated, leading to lower brain-to-plasma ratios of its substrates [26]. These findings were further confirmed in TAA-induced ALF mice [28]. Moreover, TAA-induced chronic liver failure also downregulated the expression and function of P-GP and upregulated the expression and function of Mrp2 at the BBB of rats, although BBB integrity of chronic liver failure rats was impaired [27]. However, CCl_4_-induced ALF was reported to suppress the function of P-GP in brain of rats without affecting the expression of P-GP [74]. Similarly, partial hepatectomy increased BBB permeability to cyclosporine A in mice due to suppression of P-GP function, although expressions of Mdr1a/1b mRNA in brain were not altered [75].

In general, liver failure is accompanied by increases in levels of plasma ammonia, bilirubin or bile acid, which seems to become predisposing factors for alterations in the expression and function of these ABC transporters in brain. Although liver failure is often characterized hyperammonemia, the acute hyperammonemia by ammonium acetate was reported to induce rather than decrease the expression and function of P-GP and Mrp2 in brain of rats, accompanied by activation of nuclear factor-κB (NF-κB) p65 in brain of rats [73]. In vitro data, from primarily cultured rat brain microvessel endothelial cells (rBMECs), demonstrated that the expression and function of P-GP and Mrp2 in rBMECs were significantly increased after incubation with NH_4_Cl (5 mM) for 24 h, with NF-κB pathway activation. The alterations induced by ammonia were reversed by NF-κB inhibitor BAY117082, indicating that hyperammonemia increased the expression and function of P-GP and Mrp2 at BBB via activating NF-κB pathway [73]. Chronic hyperammonemia induced by feeding with diet containing ammonium acetate plus partial portal vein ligation also significantly increased P-GP function, accompanied by increases in levels of extracellular-regulated protein kinase 1/2 (ERK1/2) phosphorylation, and reactive oxygen species (ROS) at brain of rats [unpublished]. Western blotting analysis demonstrated that chronic hyperammonemia plus partial portal vein ligation enhanced the translocation of P-GP from cytoplasm to plasma membrane. Moreover, 72-h incubation with 1 mM ammonia or H_2_O_2_ increased membrane expression and function of P-GP, ROS generation and phosphorylated ERK1/2 in HCMEC/D3 cells, which were reversed by ROS scavenger *N*-acetylcysteine and ERK1/2 inhibitor U0126, but not NF-κB inhibitor BAY117082[unpublised]. However, these results did not explain the downregulated expression and function of P-GP at the BBB under ALF, indicating the existence of other mechanisms. It was reported that the function of P-GP was suppressed in brain of CCl_4_-induced ALF rats without change of P-GP protein level [74]. In line, the plasma from the CCl_4_-induced ALF rats also suppressed the function of P-GP in Caco-2 cells [76]. Pre-incubation with plasma obtained from hepatectomized mice also enhanced the accumulation of rhodamine 123 in mouse brain endothelial cells (MBEC4), compared with sham-operated mice; even though the levels of mdr1a/mdr1b mRNA in cerebral capillaries from partial hepatectomized and sham-operated mice were similar [75]. We once reported that 10% serum from TAA-induced ALF rat slightly affected the uptakes of rhodamine 123 and sulfobromophthalein in rBMECs, and Caco-2 cells [26]. Concentrations of rat serum higher than 10% were not used because of their damage to cells. These results indicated the differences of expression and function of P-GP under various ALF models due to different mechanisms. Effects of several bile acids (cholic acid, deoxycholic acid, lithocholic acid, chenodeoxycholic acid, and ursodeoxycholic acid) and unconjugated bilirubin (UCB) on P-GP function and expression in HCMEC/D3 and MDCK-BCRP cells were investigated. The results showed that only chenodeoxycholic acid downregulated P-GP expression, and function in both HCMEC/D3 and MDCK-BCRP cells in a concentration-dependent manner [28]. These results indicated that increased chenodeoxycholic acid might be partly attributed to the downregulation of P-GP function and expression in brain of TAA-induced ALF rats and mice. Further investigations are needed to elucidate the contributions of other factors such as proinflammatory cytokines.

Unlike P-GP, both TAA-induced ALF and acute hyperammonemia induced by intraperitoneally injected ammonium acetate significantly downregulated the expression and function of BCRP, accompanied by marked increases of ERK1/2 phosphorylation and ROS at BBB of rats [53]. In MDCK-BCRP cells and rBMECs, it was found that NH_4_Cl (5 mM) and H_2_O_2_ strikingly downregulated the expression and function of BCRP. This is accompanied by remarkably increases in the levels of phosphorylated ERK1/2 and ROS. ROS scavenger N-acetylcysteine and ERK1/2 inhibitor U0126 restored the alterations by ammonia and H_2_O_2_. These results indicated that ALF down-regulated the expression and function of brain that BCRP is partly via ammonia-ROS-ERK1/2 pathway [53].

Hyperbilirubinemia is also a feature of ALF. In liver failure rats induced by bile duct ligation (BDL), it was found that BDL rats exhibited progressive decline in liver function and hyperbilirubinemia from day 3 to day 14, following BDL. In the brain tissues of BDL rats, both the function and protein levels of BCRP were progressively decreased along with time, but BBB integrity was intact. Furthermore, incubation with BDL rat serum significantly decreased BCRP function and protein levels in both HCMEC/D3 and MDCK-BCRP cells, indicating that some abnormal components in BDL rat serum might impair BCRP function at the BBB. BDL rats exhibited significant elevations of UCB and bile acids [72]. In vitro data showed that only incubation with 25 μmol/L UCB significantly increased the uptake of prazosin, and downregulated the expression of BCRP protein in HCMEC/D3 and MDCK-BCRP cells. Although bile acids (chenodeoxycholic acid, ursodeoxycholic acid and deoxycholic acid) concentration-dependently increased the uptake of prazosin in HCMEC/D3 cells, the expression of BCRP protein was unaltered. Moreover, the alterations in function of BCRP by bile acids in HCMEC/D3 cells were not confirmed in MDCK-BCRP cells. Hyperbilirubinemia rats were developed by intravenous injection of UCB. The results demonstrated that hyperbilirubinemia downregulated BCRP expression and function at BBB of rats. Correlation analysis revealed that the level of UCB negatively correlated with BCRP expression in the brains of BDL rats and hyperbilirubinemia rats. Two types of cells tested in vitro further demonstrated that UCB elevation in plasma of BDL rats contributed to the impaired function, and expression of BCRP at the BBB of BDL rats [72].

Transport of phenobarbital across the BBB is mediated by P-GP. TAA-induced ALF was reported to significantly increase brain concentration of phenobarbital, and brain-to-plasma concentration ratios of phenobarbital in mice—which was in consistence with the reduction of P-GP function and expression [28]. The ALF mice showed a significantly larger duration and shorter latency in the loss of righting reflex by phenobarbital, which was in line with increases in phenobarbital distribution of phenobarbital [28]. Similarly, cyclosporin A, a P-GP substrate, was reported to markedly aggravate harmine-induced tremors in sham-operated mice and partially hepatectomized mice at postoperative day 1 and 3, but partial hepatectomy showed stronger effect. In particular, at postoperative day 1, partial hepatectomy significantly augmented harmine-induced tremors by 53% compared with sham operation in cyclosporin A-treated mice. Further study showed that the increased effects of cyclosporine A were in line with the increased brain uptake of cyclosporine A and decreased P-GP function in brain [75], indicating the possibility that cyclosporine A increases the risk of neurotoxicity at an early phase of liver transplantation.

## 5. Future Perspectives

**Figure 3:**
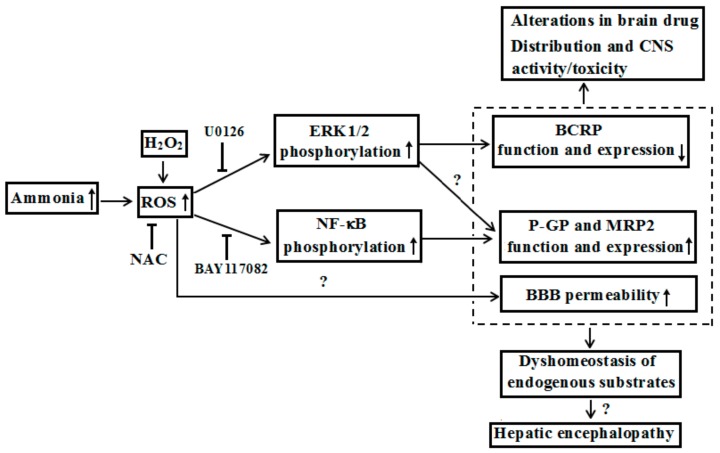
Possible mechanisms that hyperammonemia altered expression and function of ABC transporters at BBB under liver failure and its contributions to hepatic encephalopathy.

Based on current knowledge, we proposed that liver failure not only altered systemic pharmacokinetics of drugs, but also affected their brain distribution due to BBB alterations: Including changes of permeability as well as function and expression of ABC transporters. Those alterations may further affect activities/toxicities of those drugs towards CNS (Figure 3). It was reported that hepatitis A, B, and E viruses accounted for most ALF cases in the developing world [77]. Overall incidence of seizures in ALF patients was up to 33% [76]. Moreover, many drugs, such as antiepileptics (lamotrigine), antivirals (lamivudine, zidovudine and abacavir), antibiotic (levofloxacin), and immunomodulators (mycophenolate mofetil, sirolimus, tacrolimus and cyclosporine A) are substrates of BCRP or P-GP [78,79,80], some of which are also MRP2 substrates. These drugs may be administrated to liver failure patients [81,82,83,84] or liver transplant patients [85], indicating that the altered expression and function of ABC transporters at the BBB probably affect their pharmacological/toxic effects on CNS—although data cannot be directly extrapolated to human. It should be mentioned that the effects of liver failure on the expression and function of ABC transporters at BBB are often dependent on species of ABC transporters, brain regions, duration of liver failure and types of liver failure. Moreover, P-GP and BCRP are also highly expressed in intestine and liver and mediate excretion of their substrate drugs via intestinal wall and bile duct, demonstrating important roles in pharmacokinetics and pharmacology/toxicity of drugs [86,87]. Several reports demonstrated that liver failure downregulated the function and expression of intestinal P-GP [88], but increased the expression of hepatic BCRP and P-GP [89,90]. Thus, better understanding of alterations in ABC transporter functions and expressions under liver failure is of major pharmacological importance to the development and optimization of therapeutics.

ABC transporters also transport endogenous substrates. For instance, transport of steroid hormones (such as cortisol, corticosterone, aldosterone, and progesterone) is mediated by P-GP [91]. Some metabolites (dehydroepiandrosterone sulfate and dihydrotestosterone) of neurosteroids are BCRP substrates [92]. These neurosteroids are considered to contribute to the pathogenesis of hepatic encephalopathy by affecting γ-aminobutyric acid (GABA) ergic tone [93,94,95,96]. It was reported that GABA receptor positive allosteric modulator allopregnanolone, a metabolite of progesterone, was significantly increased in brain tissue from hepatic coma patients [95], On the contrast, GABA receptor negative allosteric modulator dehydroepiandrosterone sulfate was significantly decreased [96]. The reduction of dehydroepiandrosterone sulfate, together with concomitantly increased levels of allopregnanolone in brain would contribute to the phenomenon of “increased GABAergic tone” in hepatic encephalopathy. These findings indicate that the alterations in function and expression of brain ABC transporters under liver failure status will be implicated in the development of hepatic encephalopathy, which needs further investigation.

## Figures and Tables

**Table 1:** Comparison of MRP/Mrp mRNA levels in brain microvessel endothelial cell from five species. ++++, >10% of GLUT1; +++, >4% of GLUT1; ++, >2% of GLUT1; +, >1% of GLUT1; ±, <10% of GLUT1 and -, no detected, data were derived from reference [44].

Species	MRP1	MRP2	MRP3	MRP4	MRP5	MRP6
Human	++	-	±	+	++	+
Rat	++	±	±	++++	++++	++
Mouse	±	-	+	++++	+	+
Pig	±	-	++	+	+++	-
Cow	++	-	-	-	++++	-
